# Factors governing the response of *Pseudomonas aeruginosa* to hypothiocyanous acid

**DOI:** 10.3389/fcimb.2026.1878666

**Published:** 2026-07-17

**Authors:** Chelsey M. VanDrisse, Colin Swenson, Kristin M. Hudock, Arlene A. Stecenko, Balázs Rada

**Affiliations:** 1 Department of Genetics, Franklin College of Arts and Sciences, The University of Georgia, Athens, GA, United States; 2 Division of Pulmonology, Allergy, Critical Care and Sleep Medicine, Department of Medicine, Emory University School of Medicine, Atlanta, GA, United States; 3 Division of Pulmonary, Critical Care & Sleep Medicine, Department of Medicine, University of Cincinnati College of Medicine, Cincinnati, OH, United States; 4 Department of Pediatrics, University of Cincinnati College of Medicine, Cincinnati, OH, United States; 5 Division of Pulmonary Biology, Cincinnati Children’s Hospital Medical Center, Cincinnati, OH, United States; 6 Division of Pulmonology, Asthma, Cystic Fibrosis and Sleep, Department of Pediatrics, Emory University School of Medicine, Atlanta, GA, United States; 7 Department of Infectious Diseases, College of Veterinary Medicine, The University of Georgia, Athens, GA, United States

**Keywords:** antibacterial, hypothiocyanite, hypothiocyanous acid, oxidative stress, *Pseudomonas aeruginosa*

## Abstract

*Pseudomonas aeruginosa* remains a significant challenge to human medicine as it causes acute and chronic infections at several locations in the body, including the lung and eyes. *P. aeruginosa* is a very versatile, highly adaptable, opportunistic pathogen due to its large genome, ability to form protective biofilms and metabolic adaptation. Targeted, susceptibility-guided antibiotic therapy represents the best clinical approach to treat *P. aeruginosa* infections. However, due to the emergence of antibiotic resistance and the lack of a vaccine, it is critical to accelerate the development of novel, antipseudomonal therapies. Hypothiocyanous acid (HOSCN) occurs naturally in the human body and serves as an antimicrobial reactive oxidant in mucosal secretions, including the airway surface liquid, saliva, tears and milk. Mucosal peroxidase enzymes generate HOSCN from its substrates, thiocyanate (SCN^-^) and H_2_O_2_. HOSCN has been reported to kill *P. aeruginosa in vitro* which indicates its potential to be used as a new mechanism to target this bacterium. This review article provides a brief overview of the human clinical relevance of *P. aeruginosa* and summarizes current knowledge on the *in vitro* antibacterial efficacy and mechanism of action of HOSCN against this pathogen. This work also reviews the mechanisms by which *P. aeruginosa* attempts to adapt to the antibacterial actions of HOSCN.

## 1 Clinical relevance of *P. aeruginosa*

*Pseudomonas aeruginosa* is a major cause of human suffering. This Gram-negative, rod-shaped, aerobic bacterium is a leading cause of ventilator-associated pneumonia and septicemia, remains a formidable clinical adversary that exploits the vulnerability of the critically ill, causing over 32,000 healthcare-associated infections annually in the U.S. with mortality rates that can exceed 40% when multi-drug resistance is present (Bassetti et al., 2014; Yin et al., 2024). Carbapenem-resistant *P. aeruginosa* represents a major medical concern with high mortality risk (Canton et al., 2022; Venkatesan, 2022; Alharbi et al., 2025) and is on WHO’s list of antibiotic-resistant priority pathogens (Priority 1, critical, 2024) (Macesic et al., 2025). *P. aeruginosa* is one of the main leading causes (18%–20%) of nosocomial lung infections (Rahme et al., 1995; Silby et al., 2011; Mulcahy et al., 2014; Huber et al., 2016). Neutropenic patients are frequently infected with *P. aeruginosa* (Rolston and Bodey, 1992). Chemotherapy of cancer patients often results in *P. aeruginosa* lung infections (Carratala et al., 1998; Chatzinikolaou et al., 2000). In addition to transient infections, *P. aeruginosa* can also establish persistent lung infections, such as in patients with cystic fibrosis (CF), non-CF bronchiectasis (NCFBE) (Williams et al., 2010) or chronic obstructive pulmonary disease (COPD) (Bush et al., 2007). Chronic *P. aeruginosa* infection has been linked to more rapid progression of lung disease and mortality in NCFBE and CF (Jacques et al., 1998; Rowe et al., 2005; Talwalkar and Murray, 2016; Cramer et al., 2023).

### 1.1 Acute pneumonia

*P. aeruginosa* is an important cause of acute pneumonia, which is not covered by standard community acquired pneumonia (CAP) therapy (Metlay et al., 2019). Approximately 4% of CAPs and upwards of 15% of hospital acquired and ventilator acquired pneumonias are due to *P. aeruginosa* (Restrepo et al., 2018). The patients at highest risk for Pseudomonas pneumonia are those with: underlying structural lung disease (e.g., bronchiectasis or severe COPD), immunocompromised state, hospitalization in the last 3 months with intravenous antibiotic exposure, chronic tracheostomy or ventilator dependence, severe healthcare exposure and those with prior *P. aeruginosa* respiratory infection. The 2019 ATS/IDSA CAP guideline specifically recommends using antipseudomonal antibiotics, e.g., piperacillin-tazobactam or cefepime, in patients hospitalized within the prior 3 months who received intravenous antibiotics or in patients who have had a prior *P. aeruginosa* respiratory infection (Metlay et al., 2019). The aforementioned guidelines recommend obtaining a sputum culture in those patients with risk factors to substantiate the need for continued antimicrobial therapies against *P. aeruginosa* (Metlay et al., 2019). The clinical impact of Pseudomonas pneumonia is profound with high rates of progressive acute respiratory failure and/or concomitant sepsis/septic shock (Maraolo et al., 2026). All comers with acute Pseudomonas pneumonia have a mortality of 18 to 40% compared to 5.5% for pneumonia caused by other organisms, with the risk of 30-day mortality being over two times higher (Wijit et al., 2023).

### 1.2 Cystic fibrosis (CF)

The clinical landscape of *P. aeruginosa* lung infections in CF has changed since 2019 with the widespread use of highly effective CFTR modulator therapies (HEMT) like Elexacaftor/Tezacaftor/Ivacaftor, although this change has not been as dramatic as the improvement in lung function, weight and survival (Nichols et al., 2023; Durfey et al., 2025). It is true that the contemporary prevalence of *P. aeruginosa* among CF patients has decreased significantly since the introduction of HEMT. However, this is primarily seen in the pediatric CF population where less than 20% of children are infected with *P. aeruginosa* whereas double that number of adults are infected (Cystic Fibrosis Foundation Patient Registry 2024 Annual Data Report, 2024). This is most likely due to the addition of HEMT, coupled with continued surveillance for this organism and aggressive early eradication utilizing inhaled antibiotics (often paired with oral antibiotics) and even anti-pseudomonas intravenous antibiotics, resulting in a delay or failure to establish infection in younger patients. However, for those CF patients, mainly adults, with established *P. aeruginosa* infection HEMT is associated with a transient decrease in *P. aeruginosa* load that rebounds to levels seen prior to the initiation of HEMT (Langton Hewer et al., 2023; Alqasmi, 2024; Durfey et al., 2025). Thus, despite these substantial therapeutic preventative advances, managing chronic, established *P. aeruginosa* infections remains an immense clinical challenge, particularly for CF adults (Langton Hewer et al., 2023; Nichols et al., 2023; Cystic Fibrosis Foundation Patient Registry 2024 Annual Data Report, 2024). The mechanisms associated with persistence of *P. aeruginosa* in the CF lung are multiple. This includes transition into a highly protected mucoid phenotype; the formation of dense, impenetrable biofilms within the bronchiectatic airways; the development of multidrug resistance; and, in other more slower going strains, the development of antibiotic tolerance (Alqasmi, 2024; Jafari et al., 2026). A more newly recognized potential mechanism of the resilience of this organism is the role of chronic, neutrophil-dominated airway inflammation as it has been shown that *P. aeruginosa* persists in adults on HEMT only in airways where neutrophilic inflammation persists (Durfey et al., 2025). Thus, this resilience necessitates ongoing research into novel, anti-infective and anti-inflammatory strategies to combat permanent *P. aeruginosa* infection in aging CF populations who remain vulnerable to progressive respiratory decline (Jafari et al., 2026).

### 1.3 Non-cystic fibrosis bronchiectasis (NCFBE)

*P. aeruginosa* is one of the most clinically important airway pathogens in NCFBE. Chronic infection is reported in roughly 20–30% of NCFBE patients across cohorts, making it one of the most common organisms isolated in this population (Kwok et al., 2021). Its presence is consistently associated with markers of more severe disease: patients with *P. aeruginosa* tend to have worse lung function, more extensive radiographic involvement, and a higher risk of exacerbations and hospitalizations (Wang et al., 2021). Importantly, infection patterns in NCFBE are variable — ranging from transient to chronic infections or strain replacement; reflecting a less predictable natural history than in CF.

Compared with CF, the understanding of *P. aeruginosa* in the pathophysiology of NCFBE is less defined. For example, NCFBE lacks consistent definitions of “chronic infection,” optimal eradication strategies, infection control guidelines for those deemed infected and clear evidence guiding long-term antibiotic use (Conceicao et al., 2024). Key knowledge gaps in NCFBE include the causal versus associative role of *P. aeruginosa* in disease progression, the benefits, timing, and specific regimen of eradication therapy, strain-specific virulence differences, and the role of the airway microbiome in modulating outcomes.

Significant uncertainty remains regarding the treatment of *P. aeruginosa* in NCFBE, where there is limited evidence and practical challenges. Although international guidelines recommend attempting eradication at first isolation, the supporting evidence base is weak and largely derived from small observational studies rather than robust randomized trials. Based on this limited evidence, reported eradication success rates are approximately 40% at 12 months (Conceicao et al., 2024). In contrast to CF Foundation guidance (Mogayzel et al., 2014), there is no universally accepted eradication regimen in NCFBE, with wide variation in the use of oral, intravenous, and inhaled antibiotics. Additionally, while evidence for chronic suppressive therapies (i.e., inhaled or oral cycled antibiotics) was mixed, with some studies showing reductions in bacterial load or exacerbations but limited impact on lung function or quality of life (Fjaellegaard et al., 2017; Hull et al., 2025), more recent reports indicate that there are significant improvements (Guan et al., 2023; Hull et al., 2025; Lu et al., 2026). Thus, a common theme here for CF and NCFBE bronchiectasis is that these patients who also have chronic *P. aeruginosa* infections have heterogeneous microbiota and inflammatory profiles that influence their antibiotic treatment responses and therefore require more effective treatment strategies for *P. aeruginosa* (Hull et al., 2025).

Beyond efficacy and standardization, key concerns include antibiotic resistance, treatment burden, and uncertainty about long-term outcomes. Repeated or prolonged antipseudomonal antibiotic exposure —often necessary due to persistent infection— raises the risk of multidrug-resistant *P. aeruginosa*, complicating future management and limiting therapeutic options, particularly in the outpatient setting. Furthermore, it remains unclear which patients truly benefit from aggressive eradication versus suppressive strategies, and whether early treatment alters long-term disease trajectory. NCFBE lacks clear evidence or consensus on optimal timing, duration, and patient selection. These gaps underscore the need for randomized trials, better phenotyping of patients, and exploration of novel therapies targeting *P. aeruginosa* to improve outcomes in this population.

### 1.4 COPD

Chronic obstructive pulmonary disease (COPD) is a heterogeneous condition characterized by chronic respiratory symptoms and persistent airflow limitation, often resulting from significant exposure to noxious particles or gases. COPD remains a leading global health challenge with an estimated 213.4 million prevalent cases worldwide, notably exhibiting the highest age-standardized prevalence rates in North America (Cao et al., 2026). Within this population, *P. aeruginosa* has emerged as a critical pathogen, particularly in patients with advanced disease or structural lung damage (Nickerson et al., 2024). The global prevalence of *P. aeruginosa* colonization in stable COPD patients is approximately 5.6%, though isolation rates increase dramatically to between 12.4% and 18% during acute exacerbations (Kwok et al., 2025). Clinically, the presence of *P. aeruginosa* is a marker of severe disease activity; it is independently associated with an increased frequency of moderate-to-severe exacerbations and a higher risk of pneumonia (Kwok et al., 2025). Furthermore, chronic *P. aeruginosa* infection often involves the development of mucoid strains and biofilms, which significantly hinder bacterial eradication and accelerate the decline of lung function (Engler et al., 2012). The impact on survival is stark, as patients harboring multi drug-resistant *P. aeruginosa* strains face a two-year mortality rate of 60%, compared to 28% in those without such resistance (Engler et al., 2012). Consequently, *P. aeruginosa* identification is vital for risk stratification in COPD, as it serves as an independent predictor of prolonged hospital stays and increased in-hospital mortality (Usman et al., 2026).

## 2 Antimicrobial reactive oxidants

**Figure 1:**
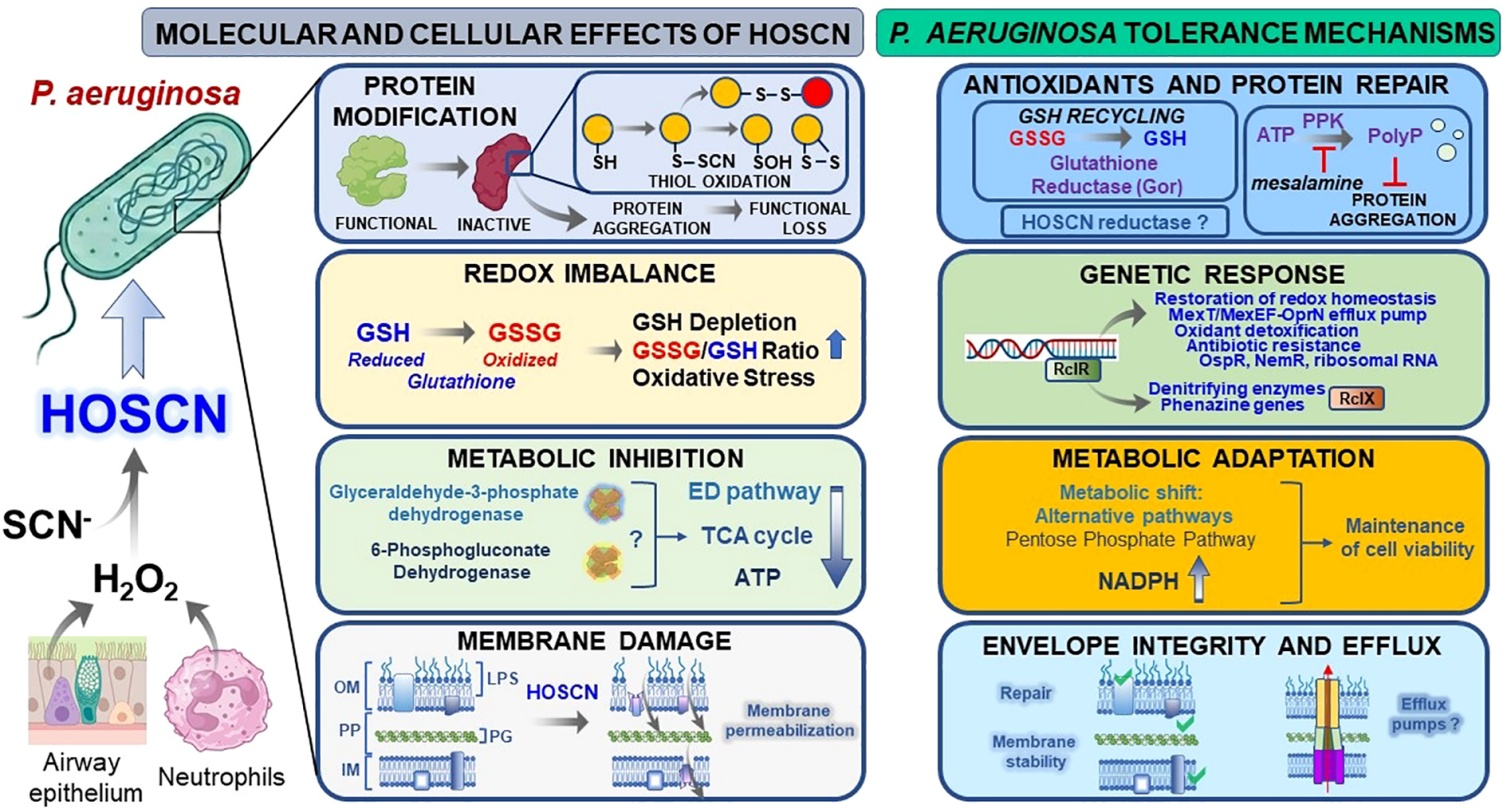
Proposed molecular and cellular mechanisms underlying the antibacterial action of HOSCN against *P. aeruginosa* and bacterial tolerance. Upon exposure of *P. aeruginosa* to HOSCN (produced at the respiratory mucosa by airway peroxidases, SCN^-^ and H_2_O_2_), several molecular and cellular changes are thought to take place in the bacterium. HOSCN targets its primary molecular target, thiol groups, in several proteins, leading to thiol oxidation, disulfide bond formations, potential protein aggregation and loss of protein function. *P. aeruginosa* protects itself against HOSCN-induced protein aggregation by polyphosphate synthesis (PolyP) by PPK, a process inhibited by mesalamine. HOSCN oxidizes GSH to GSSG, leads to GSH depletion and oxidative stress. *P. aeruginosa* likely counters this by reducing oxidized GSSG to GSH and by producing more GSH. Redox-dependent transcriptional changes also characterize the action of HOSCN, which is partially mediated by RclR. HOSCN likely inhibits the activity of key metabolic enzymes, leading to the inhibition of the major metabolic pathway using glucose [Entner-Doudoroff (ED) pathway], the tricarboxylic acid (TCA) cycle and ATP generation. As part of its metabolic adaptation to HOSCN, the bacterium shifts to alternative pathways to ensure sufficient energy and redox equivalent generation. By targeting membrane proteins, HOSCN likely leads to membrane destabilization and permeabilization, which is buffered by membrane repair and efflux pump mechanisms in *P. aeruginosa*.

The innate immune system uses reactive oxygen species (ROS) to protect itself against infections. The main ROS with antimicrobial properties are hydrogen peroxide (H_2_O_2_), hypochlorous acid (HOCl) and hypothiocyanous acid (HOSCN) (Klebanoff et al., 2013). H_2_O_2_ is less effective in its direct antimicrobial action than the other hypohalous acids (HOX) (McKenna and Davies, 1988; Love et al., 2016). H_2_O_2_ is required for the formation of hypohalous acids from their halide (Cl^-^) or pseudohalide (SCN^-^) substrates which is mediated by haloperoxidases (Davies, 2011; Winterbourn et al., 2016). The two peroxidases most relevant to airway defense are lactoperoxidase (LPO) and myeloperoxidase (MPO) (Wijkstrom-Frei et al., 2003; Klebanoff et al., 2013) (**Figure 1**). LPO is a heme-containing enzyme primarily found in milk, saliva, tears and airway surface liquid (Gerson et al., 2000). LPO is synthesized and secreted by goblet cells and submucosal glands in the airways and represents a major constituent of the airway surface liquid, accounting for approximately 1% of its total soluble protein content (Gerson et al., 2000; Klebanoff et al., 2013; Ozhan et al., 2025). MPO is a highly cationic heme protein characterized by its green color in sputum (Klebanoff, 2005). MPO is abundantly expressed in neutrophils and, to a lesser extent, in monocytes (Klebanoff et al., 2013). MPO synthesis takes exclusively place in the myeloid lineage, starting in the bone marrow during early (promyelocyte and promonocyte) stages of myelopoiesis (Hansson et al., 2006). The precursor of MPO, apoproMPO, is subjected to N-glycosylation and heme incorporation in the endoplasmic reticulum yielding an active proMPO, which is further processed in the Golgi and transported into the granules in mature neutrophils (Hansson et al., 2006). Neutrophils are the main source of MPO, storing the peroxidase in their azurophilic granules (Klebanoff, 2005). In the airways, MPO chiefly originates from activated neutrophils recruited to the sites of inflammation. Neutrophils can deliver their granule-stored MPO to the phagosome by phago-lysosome fusion or to the extracellular environment by numerous, potential mechanisms such as formation of neutrophil extracellular traps (NETs), degranulation or necrosis. LPO and MPO both use H_2_O_2_, but these peroxidases differ markedly in their substrate specificity and physiological chemistry. LPO demonstrates relatively narrow substrate specificity, with thiocyanate (SCN^-^) as its principal physiological substrate, producing hypothiocyanite (OSCN^-^) (Ozhan et al., 2025). Alternatively, urate has been shown to compete with SCN^-^ under certain conditions (Seidel et al., 2014). In contrast, MPO demonstrates broad substrate specificity (Furtmuller et al., 1998). MPO can oxidize Cl^-^, Br^-^, I^-^ and SCN^-^ to their corresponding hypohalous acids. Under physiological conditions, Cl^-^ is the dominant MPO substrate in neutrophils (Furtmuller et al., 1998). MPO and LPO overlap in their abilities to oxidize SCN^-^ (and to a lesser extent Br^-^ and I^-^), but MPO processes a wider range of anions and forms more powerful oxidants such as HOCl, compared to LPO (Furtmuller et al., 1998; Klebanoff, 2005) (**Figure 1**). Obviously, both peroxidases require H_2_O_2_ for their activities to generate antibacterial hypohalous acids. H_2_O_2_ could be generated by microbes themselves or host cells. NADPH oxidases, a family of ROS-producing enzymes, are thought to be the main sources for the HOX-forming activities of peroxidases (Rada and Leto, 2008). In neutrophils, Nox2 represents the main subunit of the NADPH oxidase complex that generates massive amounts of superoxide anions, as the primary ROS (Rada and Leto, 2008). In airway epithelial cells, Dual oxidase 1 and 2 (Duox1, Duox2) are the main H_2_O_2_ sources fueling the antimicrobial actions of airway peroxidases (Geiszt et al., 2003) (**Figure 1**).

This review article does not discuss the biological roles of HOCl, H_2_O_2_ or any other ROS in detail; it solely focuses on the effects of HOSCN in *P. aeruginosa* and bacterial anti-HOSCN countermeasures *in vitro*. Several excellent, recent, review and research articles focus on the effects of other ROS on *P. aeruginosa* (Learn et al., 1987; Hassett et al., 1999; Groitl et al., 2017; Rocha et al., 2021; Robins et al., 2025).

### 2.1 Hydrogen peroxide (H_2_O_2_)

H_2_O_2_ is being used as a disinfectant that is capable of effectively killing planktonic and biofilm cultures of *P. aeruginosa* at high enough doses *in vitro* (Boateng et al., 2011; Lineback et al., 2018). H_2_O_2_ can also be combined with “potentiators” (such as catalysts, electrochemical scaffolding, or light-based therapies) for increased *P. aeruginosa* killing (Boateng et al., 2011; Santiago et al., 2016; Abreu and Vilar, 2017; Yang et al., 2019). *In vivo* H_2_O_2_ also serves as a significant environmental stressor, often encountered during the “oxidative burst” from neutrophils, in the vicinity of airway epithelial surfaces due to Duox activity or through competition with other microbes. H_2_O_2_ can be generated in the host by NADPH oxidases or as a product of biochemical processes such as redox reactions (Sen and Imlay, 2021). H_2_O_2_ could also be generated intracellularly by the bacterium itself, since *P. aeruginosa* forms small amounts of ROS by its own respiratory chain, even if no large amounts of H_2_O_2_ are produced as primary metabolic products, unlike other bacteria (Diggle and Whiteley, 2020). H_2_O_2_ is generated by spontaneous or MPO-mediated dismutation of O_2_^.-^ in the neutrophil phagosome (Winterbourn et al., 2006). While H_2_O_2_ is capable to permeate bacteria, its direct microbicidal action has been questions at concentrations generated in the neutrophil phagosome (Imlay and Linn, 1986).

### 2.2 Hypochlorous acid (HOCl)

Under homeostatic conditions, HOCl and its conjugated base, the hypochlorite anion (OCl^–^), represent a powerful oxidizing redox system (Boecker et al., 2023). HOCl is considered to be the more effective antimicrobial agent based on their half-cell oxidation-reduction potentials and the improved penetrance of bacterial membrane by HOCl, an uncharged molecule (Snell et al., 2022; Boecker et al., 2023). At physiologically relevant pH of 7.4-7.5, HOCl and OCl^-^ are present at roughly equivalent concentrations (Boecker et al., 2023). HOCl represents a very aggressive type of ROS and has been shown to kill/inactivate most microorganisms *in vitro* (Boecker et al., 2023; Winter et al., 2025). The high oxidation-reduction potential of HOCl allows it to penetrate cell walls and membranes to exert lethal oxidative effects on DNA, proteins, and lipids (Winter et al., 2025). HOCl has been demonstrated to kill *P. aeruginosa* planktonic and biofilm cultures *in vitro* (at concentrations of 0.01-1.5%) (Romanowski et al., 2018; Herruzo and Herruzo, 2020).

Within biological systems, HOCl is characterized as the most potent and prevalent endogenous oxidant (Winter et al., 2025). The main biological source of HOCl in mammalian systems are neutrophils that generate it via the functional partnership between two redox enzymes: the NADPH oxidase and MPO (Thomas et al., 1988; Rada et al., 2008a). The NADPH oxidase first forms superoxide anions from molecular oxygen in the neutrophil phagosome that next dismutates to H_2_O_2_ (Rada et al., 2008a). MPO uses this H_2_O_2_ to oxidize its main substrate Cl^-^ to HOCl (Rada et al., 2008a). In the restricted niche of the neutrophil phagosome, it has been estimated that superoxide concentrations reach 20 μM while that of MPO rises to millimolar levels (Winterbourn and Kettle, 2013). HOCl was detected in the neutrophil phagosome with specific probes and demonstrated to react with phagocytosed microbes (Winterbourn and Kettle, 2013). The levels of phagosomal HOCl formed should be sufficient for microbial killing (Winterbourn and Kettle, 2013). While phagosomal HOCl certainly reacts with and kills engulfed bacteria, the majority of HOCl has been suggested to react with host proteins (Green et al., 2014). In line with this, alternative hypotheses of the role of ROS in microbial killing in the neutrophil phagosome have also emerged (Reeves et al., 2002; Ahluwalia et al., 2004).

### 2.3 Hypothiocyanous acid (HOSCN)

HOSCN is a powerful, short-lived antimicrobial oxidant produced in biological systems (Hoogendoorn et al., 1977). It is an inorganic acid primarily known for its role in innate immune defense (Thomas and Aune, 1978; Ashtiwi et al., 2024). In aqueous solutions, HOSCN exists in an equilibrium defined by its pKa (approximately 5.3) (Barrett and Hawkins, 2012). HOSCN is highly unstable and short-lived in physiological environments, decomposing rapidly if not immediately utilized in redox reactions (Barrett and Hawkins, 2012). HOSCN is a selective oxidant (Ashtiwi et al., 2024). Unlike the more aggressive oxidant HOCl, it does not react indiscriminately with a wide array of biological molecules (Thomas and Aune, 1978). HOSCN is generated via the peroxidase system that requires three components: 1) peroxidase enzyme (such as LPO in saliva or MPO in neutrophils; 2) H_2_O_2_ produced by cells or bacteria; and 3) thiocyanate (SCN^-^), an anion present in human secretions (Aune and Thomas, 1977). The reaction is generally described as SCN^-^ + H_2_O_2_ + peroxidase = OSCN^-^ + H_2_O (Thomas and Aune, 1978). At acidic or physiological pH, the resulting hypothiocyanite (OSCN^-^) is protonated to form the active acid, HOSCN (Barrett and Hawkins, 2012).

## 3 Antimicrobial effects of HOSCN against *P. aeruginosa*

### 3.1 *In vitro* microbicidal effects

Several reports indicate that *P. aeruginosa* is remarkably sensitive to HOSCN-mediated killing *in vitro*, often demonstrating lower survival rates compared to Gram-positive microorganisms (Loi et al., 2023; Meredith and Gray, 2023). *Pseudomonas aeruginosa* was first reported to be killed by HOSCN *in vitro* by Reiter et al. (150 µM HOSCN) (Reiter et al., 1976). When compared to other oxidants of the innate immune system, HOCl and hypobromous acid (HOBr), HOSCN was less effective in killing *P. aeruginosa in vitro* (Groitl et al., 2017). HOSCN-mediated *P. aeruginosa* killing was only observed when the bacterial growth medium contained glucose, indicating that HOSCN mainly acts on metabolically active bacteria, while the effect of HOCl was not dependent on the presence of glucose and metabolic activity (Groitl et al., 2017). HOSCN has also been reported to kill cystic fibrosis isolates of *P. aeruginosa in vitro* (range of IC_50_: 13-124 µM/hr HOSCN) (Day et al., 2020). Interestingly, these bacterial isolates were more sensitive to hyposelenocyanite (HOSeCN), a HOSCN analog generated by peroxidases from selenocyanate (SeCN^-^) using H_2_O_2_ (Day et al., 2020). HOSCN is more effective in killing *P. aeruginosa* at slightly acidic pH (6.8), compared to 7.4 (Chandler et al., 2013). This is due to the fact that both peroxidases, LPO and MPO, have this pH optimum to generate HOSCN (Welk et al., 2009; Davies, 2011). Also, the pKa of the HOSCN/OSCN^-^ equilibrium is approximately 5.3. Lowering the pH below 5.3 significantly shifts the balance in favor of the uncharged HOSCN, which is the more potent bactericidal form (Welk et al., 2009). Under pathologic conditions such as active biofilms, localized areas of infection and inflammation, the pH drops, optimizing both the peroxidase efficiency and the production of HOSCN (Welk et al., 2009).

**Table 1** showcases all reports documenting *in vitro* antimicrobial activities of HOSCN against *P. aeruginosa*.

**Table 1:** Publications reporting *in vitro* killing of *P. aeruginosa* by HOSCN.

Bacterial strain/isolate	Source	Experimental system	Ref.
ATCC 9037	–	LPO/KSCN/glucose/glucose oxidase	(Bosch et al., 2000)
ATCC 27853™ (Boston 41501)	Blood	Human airway secretions, ± dapsone	(Wijkstrom-Frei et al., 2003)
ATCC 27853™ (Boston 41501)	Blood	Human CF and non-CF ALI airway epithelial cells +SCN^-^/LPO/H_2_O_2_	(Conner et al., 2007)
PAO1	Wound	Human and rat airway epithelial cells + SCN^-^/LPO/ATP, ± catalase, DPI	(Moskwa et al., 2007)
ATCC 10145™	–	Human non-CF ALI airway epithelial cells +SCN^-^/LPO/ATP, catalase, DPI, ascorbate	(Rada et al., 2008b)
ATCC 27853™ (Boston 41501)	Blood	Human non-CF ALI airway epithelial cells (primed with flagellin or IFN-γ) +SCN^-^/LPO, ± catalase	(Gattas et al., 2009)
PAO1	Wound	*In vitro* (per Alaxia rotocol, in combination with lactoferrin: ALX-109)	(Moreau-Marquis et al., 2015)
Clinical strains: SMC1585, SMC1587, SMC1595, SMC1596, SMC5450 and SMC5451	CF sputum (Dartmouth)		
PAO1	Pseudomonas Genetic Stock Center (East Carolina University)	*In vitro*, NaSCN/LPO/H_2_O_2_, ± catalase	(Chandler et al., 2013)
Clinical isolates: AMT0027L, AMT0105L, AMT0294L, AMT0009L, AMT0058L, AMT0145L	CF (University of Washington)		
Clinical isolates: PA-39, PA-X, PA-AMT-105, PA-N3, PA-K4, PA-&	CF sputum (National Jewish Clinical Laboratory)	*In vitro*, SCN^-^/LPO/glucose/glucose oxidase	(Day et al., 2020)
PAO1	Wound		
PAO1-LAC (ATCC 47085)	Wound	*In vitro*, SCN^-^/LPO/H_2_O_2_	(Shearer et al., 2022a)
PA14	Burn wound	*In vitro*, SCN^-^/LPO/glucose/glucose oxidase	(Farrant et al., 2020)
Clinical isolates	CF airway secretions (Royal Brompton Hospital)		
PA14	Burn wound	*In vitro*, SCN^-^/LPO/H_2_O_2_	(Groitl et al., 2017)
Strain 30/70	?	*In vitro*, SCN^-^/LPO/glucose/glucose oxidase	(Reiter et al., 1976)

### 3.2 Antibacterial mechanism of action

The specific antimicrobial nature of HOSCN is defined by its targeted reactivity: thiol oxidation (**Figure 1**). Its primary biological target is the thiol group (-SH) found on cysteine residues in proteins (Lane et al., 2010). The reaction proceeds via a two-electron oxidation mechanism, leading primarily to reversible thiol modifications, which distinguishes it from the more indiscriminate, damaging oxidants like HOCl (Lane et al., 2010). The reaction of HOSCN with thiols generally proceeds in two main steps under physiological conditions (pH 7.4), often resulting in the reversible inactivation of thiol-dependent enzymes (Lane et al., 2010). The thiolate anion (R-S^-^) first reacts with the sulfur atom in HOSCN, generating a sulfenyl thiocyanate intermediate (RS-SCN) and water (Barrett et al., 2012). The sulfenyl thiocyanate intermediate is frequently unstable and quickly hydrolyzes to produce a sulfenic acid (RS-OH) and SCN^-^ (Barrett et al., 2012). RS-SCN can also attack another thiol group to form a disulfide (RS-SR) and HSCN (Barrett et al., 2012). Because HOSCN selectively targets specific sulfhydryl groups, it can disrupt microbial metabolism and enzyme function without causing the collateral tissue damage often associated with less specific oxidants. By oxidizing critical proteins in bacterial membranes and metabolic pathways, HOSCN effectively halts bacterial growth (bacteriostatic) and can be bactericidal at higher concentrations (**Figure 1**). In the context of respiratory innate immunity, it acts as a primary chemical shield against inhaled pathogens such as bacteria, fungi, and viruses (Conner et al., 2007). The production of HOSCN relies on the presence of thiocyanate (SCN^-^), a pseudohalide anion found throughout the body, including the lung in high concentrations in the epithelial lining fluid. SCN^-^ is actively transported into the airway lumen by proteins like the cystic fibrosis transmembrane conductance regulator (CFTR) and pendrin (Conner et al., 2007). Once in the airway lumen, peroxidases present in the airways catalyze the oxidation of SCN^-^ using H_2_O_2_ as a co-substrate to form HOSCN (Tenovuo et al., 1986; Morgan et al., 2011). While MPO - released by neutrophils during inflammation- is traditionally associated with the production of the harsher oxidant HOCl, it also has a high affinity for SCN^-^ and can produce HOSCN when SCN^-^ levels are sufficient (Morgan et al., 2011). HOSCN is considered a “gentler” oxidant than HOCl because it specifically targets thiol groups (-SH) in microbial proteins, effectively inhibiting bacterial metabolism without causing significant damage to host tissues. This specificity makes SCN^-^ a protective agent, as it can “scavenge” HOCl and convert it into the more biocompatible HOSCN. Consequently, the peroxidase/SCN^-^/H_2_O_2_ system is a vital component of the lung’s mucosal defense, maintaining sterility while minimizing inflammatory collateral damage (Conner et al., 2007). Lethal and sublethal doses of HOCl have been previously documented to induce massive protein aggregation in bacteria that was thought to be part of its microbicidal mechanism (Winter et al., 2008; Groitl et al., 2017). Interestingly, only lethal, but not sublethal, doses of HOSCN induce protein aggregation in *P. aeruginosa* PA14 (**Figure 1**) (Groitl et al., 2017). This indicates that the proteotoxic effects of HOSCN are likely more specific and associated with its ability to promote thiol oxidation and intra- and intermolecular disulfide bond formation.

Since HOSCN/OSCN^-^ has very high reactivity with thiols, it also readily reacts with physiological concentrations of reduced glutathione (GSH) *in vitro* (**Figure 1**) (Nagy et al., 2009). In *E. coli* and *S. pneumoniae*, mutants deficient in the gene encoding glutathione reductase (Δ*gor*), the enzyme converting oxidized glutathione (GSSG) to reduced glutathione (GSH), have higher susceptibility to HOSCN (Shearer et al., 2022c; Gray, 2024). In *E. coli*, glutathione is abundant and is oxidized effectively by HOSCN (Bennett et al., 2009; Nagy et al., 2009). An *E. coli* mutant deficient in the gene encoding glutamate-cysteine ligase (*ΔghsA*), the enzyme facilitating the first, rate-limiting step of glutathione synthesis by conjugating glutamate and cysteine to form γ-glutamylcysteine (Ge et al., 2012), also demonstrated increased susceptibility to HOSCN (Gray, 2024). Unlike many Gram-negative bacteria that maintain heavily dependent or alternative thiol pathways, *P. aeruginosa* features a robust, fully functional *de novo* glutathione biosynthetic pathway (Wongsaroj et al., 2018; Michie et al., 2020). The pathway relies on two key genes: *gshA*, which encodes the previously introduced glutamate-cysteine ligase, and *gshB*, which encodes glutathione synthetase (Wongsaroj et al., 2018). These enzymes sequentially catalyze the synthesis of GSH from glutamate, cysteine, and glycine (Shearer et al., 2022c). Wild-type *P. aeruginosa* strains (such as PAO1) produce substantial intracellular levels of GSH (Van Laar et al., 2018). While glutathione likely provides some protection to *P. aeruginosa* against HOSCN, this topic has not been specifically studied in *P. aeruginosa* in great detail, and it also has to be considered that main differences exists among bacteria in glutathione synthesis, metabolism and uptake (**Figure 1**).

In Gram-positive bacteria, the enzyme glyceraldehyde-3-phosphate dehydrogenase (GAPDH), glucose-6-phosphate dehydrogenase, aldolase and 6-Phosphogluconate dehydrogenase have been shown to be inactivated by HOSCN (Hawkins, 2009; Barrett et al., 2012; Loi et al., 2023). The concurrent inhibition of these essential bacterial enzymes by HOSCN likely triggers a multi-system collapse of bacterial metabolism, primarily decimating ATP (energy) and NADPH (reducing power) production (Hawkins, 2009). Bacteria respond by shifting their metabolism from glycolysis to other, alternative pathways, such as the pentose phosphate pathway, to maintain ATP and NADPH generation (Hawkins, 2009; Barrett et al., 2012). Whether similar metabolic changes (for instance, a shift from the Entner-Doudoroff pathway to alternate mechanisms) take place in *P. aeruginosa* upon HOSCN exposure, is very likely but remains incompletely understood (**Figure 1**).

### 3.3 Physiological relevance in humans

The concentration of SCN^-^ in the respiratory tract is substantially higher than that found in blood plasma, which typically hovers between 5-50 µM for non-smokers (Lorentzen et al., 2011; Chandler et al., 2013). The reported absolute concentration values vary significantly based on age, smoking status, and the type of airway biospecimen sampled (Lorentzen et al., 2011; Chandler et al., 2013). In undiluted tracheal and bronchial secretions of healthy adults, the absolute mean concentration of SCN^-^ is approximately 460 µM spanning an overall typical range of 30 µM-650 µM (Lorentzen et al., 2011; Chandler et al., 2013). Adult patients with CF exhibit highly variable but similarly elevated absolute concentrations in their tracheobronchial secretions, averaging roughly 350 µM – 400 µM (Lorentzen et al., 2011). In CF patients, higher endogenous SCN^-^ concentrations correlate with significantly better lung function (Lorentzen et al., 2011). The absolute SCN^-^ concentration early in life is notably lower. Diluted bronchoalveolar lavage fluid (BALF) from young children yields mean values around 280 nM (Chandler et al., 2013). When back-calculated using standard urea dilution factors to estimate the true, undiluted ASL concentration, values are estimated at roughly 28 µM – 56 µM (Lorentzen et al., 2011).

Nasal secretions demonstrate even higher values of SCN^-^ concentration. Mean absolute SCN^-^ concentrations of 400 µM were reported, with a broader upper-bound range extending from 100 µM – 1,200 µM (Lorentzen et al., 2011; Chandler et al., 2013; Meredith et al., 2022). No statistical difference in baseline nasal SCN^-^ was observed due to CFTR mutation status (Lorentzen et al., 2011).

Quantifying the absolute, *in vivo* concentrations of HOSCN/OSCN^-^ in human airway biospecimens presents an analytical challenge in biomedical literature. Because it doesn’t diffuse far from its site of generation before reacting and could react with a wide variety of compounds, there are no firmly established, direct baseline absolute concentrations for human lung ASL reported *in vivo* (Meredith and Gray, 2023). In freshly collected human saliva, early measurements reported a median OSCN^-^ concentration of 10 µM (Thomas et al., 1980).

Absolute quantification of HOSCN generation has been reliable in a variety of *in vitro* systems that contain a peroxidase, hydrogen peroxide and SCN^-^ (Hoogendoorn et al., 1977; Thomas et al., 1980; van Dalen et al., 1997; Gingerich et al., 2020). Under optimal conditions, the conversion of H_2_O_2_ to OSCN^-^ approaches a 1:1 stoichiometric yield (nearly 100% conversion efficiency before self-decomposition dominates) (Thomas et al., 1980). Upon a single addition of H_2_O_2_, *in vitro* OSCN^-^ concentration values of 50-400 µM can be achieved while higher values of >1 mM can be reached when repeated H_2_O_2_ supplementation is applied (Thomas et al., 1983; Pruitt et al., 1988; Ashby et al., 2009).

## 4 General resistance mechanisms of *P. aeruginosa* against therapies.

### 4.1 Quorum sensing

Quorum sensing, first defined in the early 1990’s through studies of virulence gene regulation in *P. aeruginosa* (Gambello and Iglewski, 1991; Fuqua et al., 1994), represents a cell-to-cell communication mechanism that enables bacteria to coordinate gene expression in a cell density–dependent manner (Miller and Bassler, 2001). Through the production and detection of diffusible signaling molecules, individual cells collectively regulate behaviors that are critical for adaptation to complex environments such as host niches (Whiteley et al., 2017). These autoinducer molecules bind to transcription factors, which then induce genes required for community survival, such as virulence, antibiotic defense mechanisms, and biofilm formation (Song et al., 2019). One of the best-characterized quorum sensing regulators in *P. aeruginosa* is the global regulator, LasR, that responds to the autoinducer *N*-3-oxododecanoyl-homoserine lactone by inducing the expression of numerous virulence genes (Pearson et al., 1994). In addition to the LasR system, *P. aeruginosa* uses interconnected quorum sensing circuits, including the RhlR/RhlI and PQS systems, which further refine gene regulation and contribute to antibiotic tolerance (Lee and Zhang, 2015). The Rhl system regulates biofilm maturation and production of rhamnolipids that can alter membrane properties and reduce antibiotic penetration (Davey et al., 2003), while the PQS system promotes outer membrane vesicle formation, oxidative stress responses, and persister cell development (Mashburn and Whiteley, 2005). Together, these systems form a hierarchical and partially redundant regulatory network that enhances bacterial survival during antibiotic exposure (Schutz and Empting, 2018; Bru et al., 2019). Consistent with this, mutations in *lasR* are frequently found in a myriad of hyper-virulent *P. aeruginosa* clinical isolates (Smith et al., 2006). However, even with LasR absent, quorum sensing activity and virulence are often maintained through alternative pathways, underscoring the robustness of this regulatory mechanism.

### 4.2 Biofilms

What distinguishes *P. aeruginosa* from other pathogens with regards to antibiotic survival, is its ability to form robust biofilms within host environments (Costerton et al., 1999). While a myriad of pathogens encode enzymes that directly neutralize antibiotics, *P. aeruginosa* biofilms confer tolerance through a combination of metabolic, physiological, and physical mechanisms that collectively enable survival in the presence of antibiotics (Stewart and Costerton, 2001). Biofilm formation is induced through surface sensing and quorum sensing, driving the transition from planktonic to surface attached or suspension biofilm growth (O'Toole and Kolter, 1998). During early biofilm development, subpopulations of cells undergo programmed lysis, releasing essential components of the biofilms such as extracellular DNA (eDNA) (Whitchurch et al., 2002), while others produce exopolysaccharides such as Psl, Pel, and alginate (Colvin et al., 2012). These components make up an extracellular matrix, which encapsulates and protect cells, while also generating steep gradients in oxygen, nutrients, and redox state, resulting in metabolically heterogeneous subpopulations (Stewart and Franklin, 2008). These gradients limit antibiotic efficacy both by restricting penetration and by generating slow-growing or dormant cells that are inherently less susceptible to antibiotics (Stewart et al., 2015). Oxygen limitation within biofilms promotes anaerobic respiration and reduced proton motive force, further decreasing the uptake and efficacy of antibiotics such as aminoglycosides (Borriello et al., 2004). The biofilm matrix itself can also directly interact with antimicrobial agents, such as cationic antibiotics, that are sequestered by eDNA in the biofilm (Mulcahy et al., 2008). As a result, *P. aeruginosa* biofilms can tolerate antibiotic concentrations that are 10- to 1,000-fold higher than those required to inhibit planktonic cells (Mah and O'Toole, 2001). Others have successfully targeted biofilm-specific metabolic processes as a promising therapeutic strategy. For example, addition of molecules that inhibit anaerobic respiration sensitizes biofilm cells to clinical antibiotics such as ciprofloxacin (Spero and Newman, 2018; Kim et al., 2024). Additionally, phenazines are redox-active metabolites that support biofilm metabolism under oxygen limitation (Glasser et al., 2014), and their degradation enhances susceptibility to tobramycin (Costa et al., 2017; VanDrisse et al., 2021). These thick, matrix-encased biofilms physically shield bacterial colonies within the thick mucus of the patient’s airways, allowing slow-growing “persister cells” to survive otherwise lethal drug concentrations (Moradali et al., 2017). Persister cells represent phenotypic variants that achieve tolerance primarily by shutting down their metabolic machinery and entering a state of dormancy (Alharbi et al., 2025). When the antibiotic pressure is removed, these cells resume normal growth, and give rise to a new population that is once again completely sensitive to the original antibiotic (Alharbi et al., 2025). Consequently, the clinical trajectory of infected patients shifts dramatically, as infections evolve from treatable acute episodes into incurable, chronic pulmonary infections that induce extensive tissue destruction and drive high mortality rates (Lyczak et al., 2002; Pang et al., 2019). Together, these approaches highlight how biofilm-specific physiology can be exploited to overcome antibiotic tolerance in *P. aeruginosa* infections.

### 4.3 Antimicrobial resistance

*P. aeruginosa* remains one of the most severe public health challenges due to its exceptional adaptability and skyrocketing rates of antimicrobial resistance, particularly in respiratory infections (Lyczak et al., 2002; Moradali et al., 2017). Surveillance data highlighting clinical isolates from respiratory infections reveals staggering resistance rates, with global meta-analyses demonstrating nonsusceptibility in up to 94.9% of respiratory strains to at least one primary antimicrobial agent (Jafari et al., 2026). The pathogen has acquired robust resistance across nearly all major antipseudomonal classes, specifically targeting beta-lactams, third-generation cephalosporins (like ceftazidime), and fluoroquinolones (Pang et al., 2019; Jafari et al., 2026). Most alarming is the rapid surge in carbapenem-resistant *P. aeruginosa* isolates, which neutralize critical last-resort treatments like imipenem and meropenem through mechanisms like carbapenemase production and porin channel loss (Pang et al., 2019; Jafari et al., 2026). The primary medical dilemma driving treatment failure with *P. aeruginosa* stems from its multi-layered defense architecture, which integrates intrinsic mechanisms like extremely low outer membrane permeability and active multidrug efflux pumps with adaptive traits like biofilm formation (Moradali et al., 2017; Pang et al., 2019).

### 5 Antioxidant shield of *P. aeruginosa*

*P. aeruginosa* employs a sophisticated hierarchical antioxidant defense system to neutralize ROS encountered during the neutrophil-mediated oxidative burst or airway epithelial surface exposure. At the forefront of this defense are superoxide dismutases, SodA (manganese-dependent) and SodB (iron-dependent), which catalyze the dismutation of O_2_^.-^ into H_2_O_2_ and molecular oxygen (Gellatly and Hancock, 2013). To degrade H_2_O_2_, the bacterium utilizes three primary catalases—KatA, the major constitutive catalase, and the inducible KatB and KatE— which directly convert H_2_O_2_ into water and molecular oxygen (Mishra and Imlay, 2012; Su et al., 2014; Diggle and Whiteley, 2020; Deng et al., 2025). Furthermore, the OxyR transcriptional regulator senses oxidative stress through the formation of an internal disulfide bond, subsequently upregulating the expression of the ahpCF operon, which encodes alkyl hydroperoxide reductase for the reduction of organic peroxides. Against neutrophil-derived HOCl, the bacterium utilizes the RclR-regulated system and thiol-dependent peroxidases like Ohr to maintain redox homeostasis (Mishra and Imlay, 2012). The glutathione-independent thioredoxin system, involving TrxA and the reductase TrxB, provides essential reducing equivalents to repair oxidatively damaged proteins and support peroxidase activity. Additionally, *P. aeruginosa* produces the pigment pyocyanin, which, while often considered a toxin, can also participate in redox cycling to modulate the local oxidative environment. The bacterium utilizes proteins like Dps to sequester intracellular free iron, which prevents the formation of highly toxic hydroxyl radicals through the Fenton reaction (Diggle and Whiteley, 2020). In chronic infection states, *P. aeruginosa* often exists in biofilms, which can be up to 1,000-fold more resistant to H_2_O_2_ than planktonic cells, as the matrix slows the diffusion of oxidants (Hassett et al., 1999; Su et al., 2014). *P. aeruginosa* alginate has been shown to scavenge neutrophil-derived HOCl (Learn et al., 1987). These enzymatic defenses are coordinated by the SoxR and OxyR systems, ensuring a rapid transition to a highly resistant “antioxidant mode” upon sensing phagocyte-derived oxidants (Cui et al., 2025).

Different, protein and non-protein based, mechanisms have been identified in the past to protect bacteria from protein aggregation caused by oxidative stress (Dahl et al., 2015). The protein chaperone Hsp33 and the chemical chaperone polyphosphate (polyP) represent such main mechanisms (Winter et al., 2008; Dahl et al., 2015). Upon oxidative stress, Hsp33 becomes quickly activated to serve as a molecular chaperon (Winter et al., 2008). PolyP is generated from ATP by the enzymatic action of the polyP kinase (*ppk*), and acts by stabilizing proteins and preventing them from aggregation (Dahl et al., 2015). PolyP accumulation is also controlled by the activity of the exopolyphopsphatase (PPX), a polyP degrading enzyme (Gray, 2024). Both Hsp33 and polyP were shown to contribute to the tolerance of *P. aeruginosa* against sublethal doses of HOCl and HOBr (Groitl et al., 2017).

The robustness of this antioxidant shield represents an important reason why *P. aeruginosa* can persist in the highly inflamed, neutrophil-rich environment of the bronchiectatic lung.

## 6 Tolerance mechanisms of *P. aeruginosa* to HOSCN

Enzymes specific for disarming HOSCN referred to as HOSCN reductases have been identified in *Escherichia coli* (RclA), *Staphylococcus aureus* (MerA) and *Streptococcus pneumoniae* (Har) (Meredith et al., 2022; Shearer et al., 2022b; Shearer et al., 2023). The first identified *E. coli* HOSCN reductase RclA is a flavoprotein that had been previously implicated in reactive chlorine resistance, reduces HOSCN to SCN^-^ with near-perfect catalytic efficiency and effectively protects *E. coli* against HOSCN toxicity (Meredith et al., 2022). In *S. aureus*, the flavoprotein disulfide reductase MerA protects the bacterium from HOSCN by functioning as a HOSCN reductase (Shearer et al., 2023). Evidence suggests that MerA is under the control of the redox-sensitive repressor HypR, which becomes oxidized to intermolecular disulfides under HOSCN stress, leading to its inactivation and de-repression of MerA transcription to support tolerance to HOSCN (Shearer et al., 2023). In *S. pneumoniae*, a flavoprotein disulfide reductase with HOSCN reductase activity has been identified and named Har (Shearer et al., 2022b). While Har is dispensable for *S. pneumoniae* growth in the presence of HOSCN, bacterial growth was entirely blocked when Har deficiency was combined with disruption of GSH import into *S. pneumoniae* (Shearer et al., 2022b). While a direct, functionally identical “hypothiocyanite reductase” counterpart in *P. aeruginosa* has not been identified yet, the lysates of some *P. aeruginosa* clinical isolates demonstrated a low but detectable HOSCN metabolizing activity (Chandler et al., 2013). Interestingly, other clinical isolates and the reference strain of *P. aeruginosa* demonstrated no detectable activity to use HOSCN (Chandler et al., 2013). These results indicate that some clinical isolates of *P. aeruginosa* may have unidentified mechanisms to metabolize HOSCN (**Figure 1**).

While its antibacterial mechanism of action in *P. aeruginosa* remains incompletely understood, some data suggest that HOSCN causes widespread protein misfolding and aggregation, essentially “clumping” the internal machinery of the cell (**Figure 1**) (Groitl et al., 2017). Protein aggregation is only induced in *P. aeruginosa* by lethal HOSCN doses, but not by sublethal concentrations (Groitl et al., 2017). This is due, at least in part, to the intense polyP formation triggered by sublethal HOSCN doses as the sensitivity of the *ppk* deletion mutant of PA14 to HOSCN is drastically enhanced, compared to the parental strain (Groitl et al., 2017). This is not true for the mutant deficient in hslO, the gene encoding the Hsp33 chaperon, indicating that Hsp33 does not play a significant role in protection against HOSCN in *P. aeruginosa* (Groitl et al., 2017). Mesalamine, a prescription medication for the treatment of mild-to-moderate ulcerative colitis, has been found to inhibit polyP synthesis in bacteria, including *P. aeruginosa* (Dahl et al., 2017). Mesalamine treatment increased the sensitivity of PA14 to HOSCN, an effect that was absent in the *ppk* deletion mutant, indicating that mesalamine could provide an option to treat *P. aeruginosa* infection, particularly in combination with HOSCN (**Figure 1**) (Groitl et al., 2017).

In *E. coli*, an HOCl-reactive transcription factor has been identified, Rclr (Parker et al., 2013). In *P. aeruginosa*, RclR has been reported to also provide tolerance in response to HOSCN (and HOCl) (Farrant et al., 2020). Additionally, *rclR* is adjacent to a single-gene operon encoding a putative peroxiredoxin gene (*rclX*) (Farrant et al., 2020). Both, *rclR* and *rclX* deletion mutants of *P. aeruginosa* were susceptible to 1 mM HOSCN but only the *rclR* mutant was sensitive to 800 μM HOSCN (Farrant et al., 2020). This indicates that both genes provide some protection to HOSCN but their activities depend on the HOSCN dose (Farrant et al., 2020). The role of these genes in HOSCN protection remained when *P. aeruginosa* was grown in artificial sputum medium (Farrant et al., 2020), which mimics the CF airway environment. The expression of *rclX*, but not that of *rclR*, is increased upon exposure to HOSCN, in reference strains and clinical isolates, as well (**Figure 1**) (Farrant et al., 2020).

Two independent reports studied transcriptional changes of *P. aeruginosa* to HOSCN. In the work by Groitl B. et al., exposure to sublethal doses of HOSCN lead to transcriptional changes of genes involved in oxidant detoxification (catalase, methionine sulfoxide reductase, alkyl hydroperoxide reductase) and restoration of redox homeostasis (e.g. glutathione peroxidase and glutathione reductase) (**Figure 1**) (Groitl et al., 2017). These genes were also induced by HOCl and HOBr, so were not specific to HOSCN (Groitl et al., 2017). Out of the 70+ regulons of the bacterium analyzed, only one, MexT responded similarly to HOSCN, HOCl and HOBr, proposing that the induction of the MexT regulon is a general antioxidant response of *P. aeruginosa* (Groitl et al., 2017; Farrant et al., 2020). MexT is a redox-sensitive LysR-type transcriptional regulator that also initiates the expression of the MexEF-OprN multidrug efflux system, which removes electrophilic metabolites from the bacterium to restore the redox balance (**Figure 1**) (Fargier et al., 2012; Groitl et al., 2017; Farrant et al., 2020). The OxyR and LasR regulons did not respond to HOSCN treatment (Groitl et al., 2017). Among regulons that have not been identified in *P. aeruginosa* at the time of the study but have been shown to be responsive to HOCl in other microbes, HOSCN induced the expression of the OspR regulon that regulates glutathione peroxidase, and the NemR regulon described as an HOCl-responsive repressor in *E. coli* (Gray et al., 2013; Atichartpongkul et al., 2016). When compared between HOCl and HOSCN, the most distinct differences in induced transcriptional changes have been observed in the functional categories of molecular chaperons (Groitl et al., 2017). While 12 or 9 heat shock genes were induced by HOCl or HOBr, respectively, only two were upregulated by HOSCN (*grpE, hsIV*) (Groitl et al., 2017). On the other hand, HOSCN upregulated much higher number of genes associated with antibiotic resistance and membrane proteins, compared to HOCl and HOBr, proposing that HOSCN primarily interacts with membrane proteins (**Figure 1**) (Groitl et al., 2017).

The second, later report by Farrant K.V. et al. found that HOSCN upregulated 105 genes and downregulated 16 genes in PA14 (Farrant et al., 2020). 70% of the genes upregulated by HOSCN, were also induced by HOCl, indicating a large overlap in the bacterial response to oxidants (Farrant et al., 2020). The largest functional groups among the induced genes belong to noncoding RNA, protein secretion/export apparatus, and antibiotic susceptibility and resistance (Farrant et al., 2020). The role of RclR was also studied. RclX was the gene most strongly upregulated by RclR in response to HOSCN (Farrant et al., 2020). In the presence of HOSCN, RclR upregulated 132 genes but only 9% of those were also induced by HOCl (Farrant et al., 2020). In response to HOSCN, RclR downregulated 213 genes, but only 1% of those were also downregulated by HOCl (Farrant et al., 2020). Secreted factors represented the largest portion of the functional gene groups regulated by RclR in response to HOSCN, or HOCl (Farrant et al., 2020). Genes involved in the synthesis and regulation of the type three secretion system were also induced by both oxidants (Farrant et al., 2020). In the presence of HOSCN, RclR positively regulated the expression of phenazine genes involved in the production of the virulence factor pyocyanin and that of operons encoding denitrifying enzymes (**Figure 1**) (Farrant et al., 2020).

These groundbreaking transcriptional studies identified several mechanisms that are activated or downregulated in *P. aeruginosa* in response to HOSCN (Groitl et al., 2017; Farrant et al., 2020). Both studies revealed a connection between response to reactive oxidants and antibiotic resistance. HOSCN-dependent regulation of the MexT-dependent efflux pump provides one molecular explanation for this link and proposes that not only antibiotics but toxic by-products of the redox reactions of HOSCN could also be removed via this pump mechanisms (**Figure 1**) (Groitl et al., 2017; Farrant et al., 2020).

## 7 Open questions

While recent reports advanced our knowledge on the molecular details of the mechanism of action of HOSCN against *P. aeruginosa* and on bacterial tolerance mechanisms, several questions in this specific field remained unanswered and urge future research. Some of the most important questions to be answered are:

- Can HOSCN damage already established *P. aeruginosa* biofilm aggregates?
- Can HOSCN sensitize *P. aeruginosa* to the effects of antibiotics, bacteriophages or other, approved or developing, antibacterial clinical therapies?
- Is the antimicrobial effect of HOSCN on *P. aeruginosa* affected by the elexacaftor-tezacaftor-ivacaftor therapy in CF?
- Is the antibacterial effect of HOSCN on *P. aeruginosa* affected by brensocatib, a recently approved medication for NCFBE?
- Can identified tolerance mechanisms of *P. aeruginosa* against HOSCN be targeted pharmaceutically to enhance its antimicrobial efficacy?
- Is the antimicrobial activity of HOSCN influenced by the airway microbiome composition, properties of the mucus, biofilm architecture, or metabolic adaptation of chronic *P. aeruginosa* isolates?
